# Development of an intrinsic health risk prediction model for camera-based monitoring of older adults living alone

**DOI:** 10.1038/s41598-022-23663-2

**Published:** 2022-11-07

**Authors:** Minji Kim, Song-iee Hong, Sekyoung Youm

**Affiliations:** 1 Department of Industrial and Systems Engineering, Dongguk University, 30 Pildong-ro 1-gil, Jung-gu, Seoul, 04620 Republic of Korea; 2 Department of Social Welfare, Dongguk University, 30 Pildong-ro 1-gil, Jung-gu, Seoul, 04620 Republic of Korea

**Keywords:** Health care, Engineering

## Abstract

The number of older adults in Korea is increasing, along with the number of depressed older patients. The causes of depression in older adults include social isolation with negligible interaction with others, irregular nutritional habits, and self-negligence, i.e., they do not engage in any activity. These factors, self-negligence, social isolation, and irregular nutritional habits, are defined as inherent health risks, and in this study, we detected them. These factors can only be derived through long-term monitoring, but the current monitoring system for older adults is severely limited as it focuses only on emergencies, such as “falls.” Therefore, in this study, the goal was to perform long-term monitoring using a camera. In order to capture the physical characteristics of the older adults, the ETRI-Activity3D data were used for training, and the skeleton-based action recognition algorithm Posec3d was used. By defining 90 frames as the time taken for one action, we built a monitoring system to enable long-term monitoring of older adult by performing multiple action detection in one video. A reliable monitoring system, with 98% accuracy, 98% precision, 99% recall, and 98% F1, was successfully established for health monitoring of older adults. This older adult monitoring technology is expected to improve the quality of medical services in a medical environment as well as the objective, activities of daily living test, which does not depend on the observer through daily life detection.

## Introduction

According to Korea´s statistics on older adults in 2021, older adults comprise 16.5% of the total population in South Korea. Among them, community older adults living alone, account for 35.1%, which is expected to continue to rise in the future^1^. According to the statistical data of 2019, as the number of older adults increased, the proportion of depressed patients belonging to this category became 40.4% of the total population, and this proportion is continuously increasing. Depression in older adults has a negative impact on their overall life and may lead to death depending on the degree of depression, and prolonged depression is certainly fatal to older adults living alone^2,3,18^. Among the many factors that cause depression in older adults, social isolation and self-neglect are the most common causes^4,19^. The recent guidelines for “social distancing”^15–17^, in which people were advised to stay at home due to the COVID-19 pandemic, had an indirect effect on the onset of depression in older adults^5^. A life of isolation due to a decrease in intimacy with neighbors and a decrease in interpersonal relationships due to “social distancing” can lead to depression^19^. Further, the rate of self-neglect, which means that the older adults do not take care of themselves and are inactive, is increasing every year^20^. Self-neglect also has a significant correlation with depression^4^. In addition, older adults show irregular nutritional intake due to various factors such as economic status and loss of appetite, and an irregular nutritional intake increases the nutritional risk factors^21,22^. Although such irregular eating habits do not cause short-term health deterioration, they can worsen health in the long term. Social isolation, self-neglect, and abnormal nutritional habits can be discovered by observing daily life patterns and through the activities of daily living (ADL) assessment. ADL refers to the basic activities that an individual needs to perform in order to function independently in daily life^10^. A correlation between the ADL and the cognitive dysfunctions has been reported^11^. Since ADLs are used as indicators to measure the severity of dementia^24^ and to determine the effectiveness of dementia treatment agents^12^, ADLs can provide specific information on the ability of older adults to manage their daily life independently^13^. Currently, the ADLs are either conducted directly with older adults or through observations and interviews with their family members and nurses^13,14^. However, the reliability of such assessments is questionable as they may be affected by subjective factors^12^.

In recent times, the fourth industrial revolution is garnering considerable attention, and the development of technology to realize it has accelerated. Consequently, numerous studies are being conducted to solve the current societal problems via the convergence of the fourth industrial revolution technology^6^. Technologies for monitoring older adults have been proposed to maintain the independence of older adults, aid in decision-making, and reduce the burden on caregivers^7,40^. Further, older adult monitoring systems can help before the unexpected happens^39^. The focus of current monitoring technology is on identifying the older adults’ emergency situations^8,9^. However, a monitoring system focusing on emergencies cannot detect the general lifestyle of an older adult, such as lack of various activities and self-negligence. Several researchers have also performed non-emergency, daily life detection. Yang and Hsu monitored the rhythm of the daily life of older adults using four movement detectors and one appliance usage detector; however, they could not detect the exact action being performed^41^. Awais et al. performed daily living behavior monitoring through four sensors attached to the body. However, only four simple actions (sit, stand, walk, and lie down) were detectable^42^. Matsui et al. used several motion sensors, ambient sensors, and door sensors to monitor the daily life of the elderly, but only five behaviors (bathing, cooking, eating, going out, sleeping) could be detected with a low precision^43^. Fernando et al. performed the detection of falls and daily life behaviors of the elderly using a camera. However, only a small number of daily life categories (standing, sitting, walking, falling, and grabbing) were monitored in this study^44^. As can be seen from these previous studies, the current technology has limitations in accurately monitoring daily life, including uncertain movements, and thus, many categories of daily life cannot be detected.

Therefore, this study was performed to design a monitoring system that can automatically evaluate various categories of behaviors, such as social isolation, inactivity, and unhealthy behavior. This designed monitoring system can detect several behavioral categories together, unlike the current single monitoring systems, which can detect on life-threatening emergencies. Furthermore, instead of simple monitoring, the developed system can be applied to the data of older adults’ actual daily lives. Therefore, the study was performed in two stages: first, we attempted to advance the camera-based technology of monitoring older adults’ daily lives; second, we plan to applied this advanced skill to the real settings of older adults, who are institutionalized at nursing homes in Kang Hwa, South Korea. This article is focused on the first stage of our study. We expect this camera-based monitoring system to improve the quality of health care services. Furthermore, the monitoring system can facilitate an objective and quantitative ADL evaluation, which is dependent on observations and interviews, and ensure the safe management of older adults^40,45^.

## Methods

### Materials

**Figure 1:**
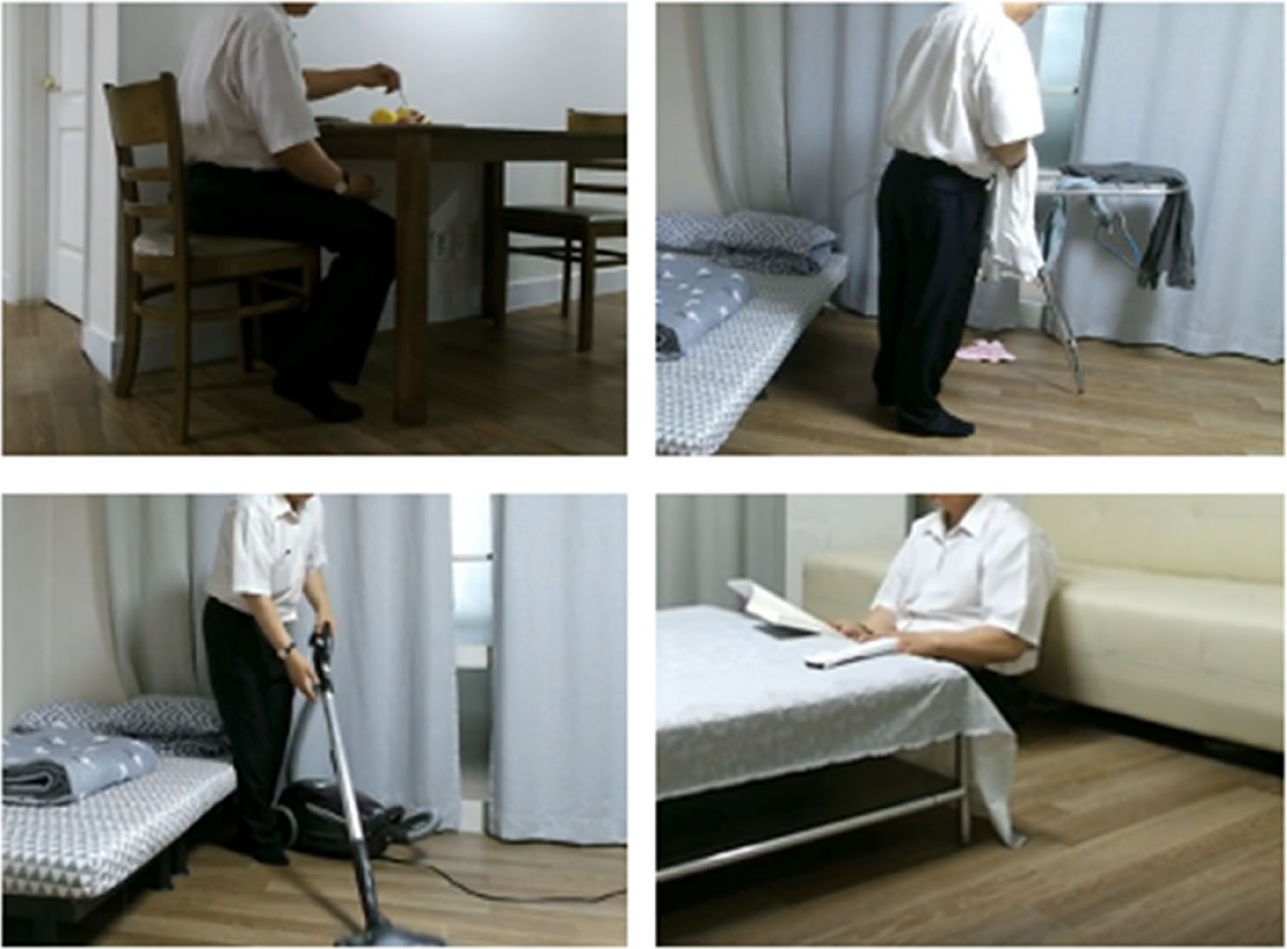
Sample of the ETRI-Acticity3D dataset.

In this study, we used the ETRI-Activity3D dataset by filming the lives of older adults from a robot’s point of view to solve the problems of an aging society and to learn the behavior of older adults^32^. The data was collected in a 102 m^2^ apartment setting reflecting a real-home environment, and it was constructed to elicit similar behaviors of older adults with maximum possible accuracy. Figure 1 shows a typical example of the video data. The actual data used from the ETRI-Activity3D dataset are shown in Table 1. The ETRI-Activity3D data were approved on April 20, 2021 after submitting an agreement on the ETRI AI Nanum website (https://nanum.etri.re.kr/share/dhkim008/robot_environment2?lang=en_KR).

**Table 1:** Definition of ETRI-Activity3D data.

Items	Content
Total number of samples	5339 (train 4091/test 1248)
Number of behavior classes	13
Number of people filmed	20 people (10 older men, 10 older women)
Filming environment	102 m^2^ apartment living environment
Filming location	Bathroom, kitchen, living room, etc
Used data format	RGB videos
FPS	25

There were a total of 55 classes in the ETRI-Activity3D dataset. However, in accordance with the behavior classes for the intrinsic risk outlined, the categories were defined as (1) behaviors that can be used to assess the ability to perform ADLs as a basic daily routine; (2) behaviors that may be unhealthy in the long term; and (3) behaviors that can indicate the social relationships of older adults. Therefore, we defined a total of 13 classes, and these classes satisfying the respective conditions are presented in Table 2. Several behavior classes were merged and redefined into a single class. The behavior of using a vacuum cleaner and that of cleaning the floor while bending forward were combined and defined as “Cleaning the room.” Similarly, reading a book and reading a newspaper were combined and defined as “Reading.” The action of making or receiving a call and the behavior of operating a smartphone were also combined and defined as “Using a phone”.

**Table 2:** Definition of behavior classes used by the developed system.

Class criteria	Behavior class	Total number of data
(1) Behaviors that can be used to assess the ability to perform ADL as a basic daily routine	Using a gas stove	266
	Cleaning the room	398
	Cleaning the furniture	286
	Hanging laundry	392
	Reading	558
	Using a remote	324
	Lying	378
(2) Behaviors that may be unhealthy to health in the long term	Eating	507
	Taking medicines	580
	Drinking	376
	Smoking	362
(3) Behaviors that can indicate the social relationships of the older adults	Talking	330
	Using a phone	582

### Action recognition

Action recognition technology is essential to monitor the daily lives of older adults and has been widely applied to video information search, daily life security, and CCTV surveillance^23^. This technology is divided into two types of actions: action classification and action detection^24^. Action classification implies classifying the type of action a person is performing in a video. Consequently, the video data used as the input should consist of an image of an action. In contrast, action detection detects which action is taken at a certain point in a video that was not cut, based on a specific class criterion^23,24^. Since most videos collected in real life are unedited videos that include multiple actions rather than a specific action, action detection is essential for recognizing the specific target actions of a person in the actual video^24,25^.

The methods of action recognition can be broadly divided into the following categories: (i) a method that uses RGB image data without changes^26,27,34^, and (ii) a method that detects an action using the skeleton coordinates of a human body derived from the RGB image data^28,29^. However, when RGB images are used, the action detection may be sensitive to information other than actions, such as background and color^19,21,22,34^. Therefore, if action recognition is performed using the skeleton coordinates of a human body in RGB images, then it is possible to overcome the limitations by solely obtaining the motion data without any influence of the background or lighting^30,31^. For this reason, Posec3d^28^, which performs skeleton-based action recognition, was used in this study.

**Figure 2:**
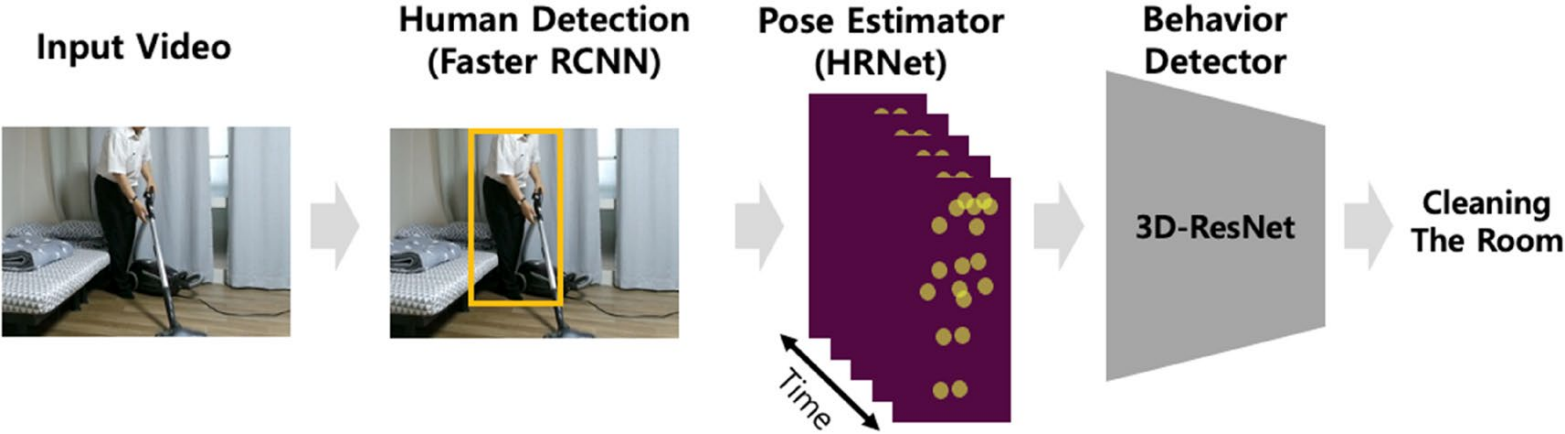
Posec3d framework^28^.

Posec3d can be divided into two main segments: a pose estimator and a behavior detector. In the pose estimator, a video, entered as the input data, is sliced into images at a certain rate (frame per second; fps), and the human skeleton coordinates are derived from each image. In this study, the pose estimator stage involved the estimation of human pose using the Top-Down method, which has an advantage over the Bottom-Up method in terms of accuracy^36^. Therefore, the human body was first detected and then the human skeleton coordinates were derived. First, the object detection algorithm faster region-based convolutional network (Faster CNN)^37^ was used to detect a person, and the pose estimation algorithm high resolution network (HRNet)^35^ was used to extract the human skeleton coordinates of the detected person. After converting each extracted human skeleton coordinate into a two-dimensional (2D) heatmap, it was stacked according to time flow to construct a 3D heatmap. The behavior detector used the 3D ResNet-based 3D CNN as the input for the 3D heatmap to detect and identify the actions performed by a person in a video. Figure 2 shows the framework of Posec3d.

## Development of the ADL monitoring system

### Framework

**Figure 3:**
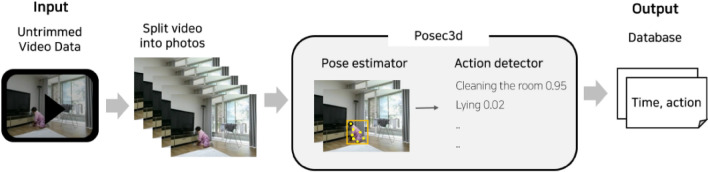
Behavior monitoring system framework.

The study framework (Fig. 3) allows older adults to record their behaviors using Posec3d, which is designed to evaluate older adults’ ADLs to detect intrinsic risks. Posec3d enables a state-of-the-art recording in the field of action recognition using human skeleton coordinates^33^.

In this study, we evaluated different behaviors of older adults, such as social isolation, self-neglect, and long-term unhealthy behaviors. These behaviors elucidated in this study are: (1) basic behaviors that can be used to evaluate the personal capacity of ADLs, (2) behaviors that can be unhealthy in the long term, and (3) behaviors that can be assessed to identify the social relationships of older adults.

### Data processing

**Figure 4:**
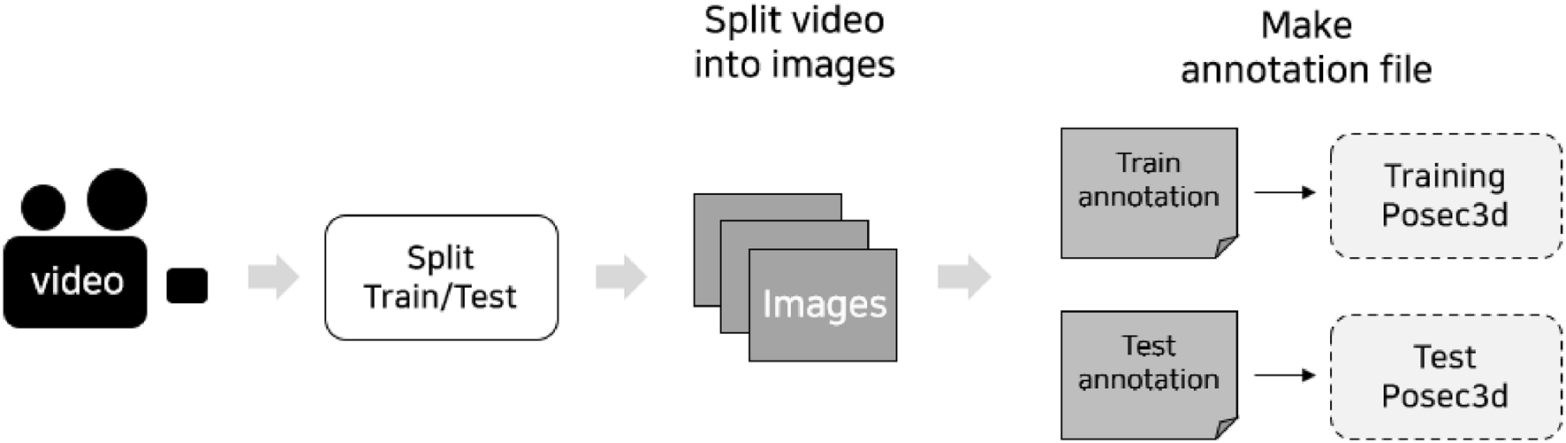
Illustration of the process of creating annotations.

All the performance evaluation processes were carried out using Python. Figure 4 shows a series of steps used for preprocessing the data. These processes created an annotation source of learning in the Posec3d algorithm.

The training and test data were divided into a ratio of 8:2 based on the number of participants and were set as the training dataset (8 males and 8 females) and test dataset (2 males and 2 females). The ETRI-Activity3D dataset was a video filmed at 25 fps and sliced to 25 fps using ffmpeg to create several images.

Annotation is a collection of information on the data to be used in the learning. In this study, the annotation contained information for each video as shown in Table 3. This information was stored in a dictionary format, and the final annotation was in the form of a list containing the annotation of each video. Here, “Frame_dir” is used to distinguish the name of the video, and “Img_shape” and “Original_shape” indicates 1080 width and 1920 height (1080, 1920). Because each video had a different length, the total number of frames also varies.

**Table 3:** Annotation definitions and examples used in the system.

Item	Explanation	Data format	Explanation on data format
Frame_dir	Name of video	String	Ex) A001_P001_G001_C004
Img_shape	Size of image data	Tuple	(height, width) = (1080, 1920)
Original_shape	Original size of video	Tuple	(height, width) = (1080, 1920)
Total_frames	Total number of video frames	Integer	Ex) 456
Keypoint	Skeleton’s x,y coordinates for people inside each frame of the video	Array	[N(number of people), T(number of frames), K(number of keypoint = 17), 2(x,y coordinates)]
Keypoint_score	Confidence value of the skeleton value of the person in each frame in the video	Array	[N(number of people), T(number of frames), K(number of keypoints = 17)]
Label	Class of the video	Integer	Ex) 0

The keypoints were extracted using HRNet^35^, and then a total of 17 skeleton coordinates were used as shown in Table 4. The order of each skeleton coordinate is specified in Table 4. Here, “Keypoint_score” indicates the confidence value of each keypoint. Therefore, if the confidence value was high, then the human skeleton coordinate could be detected with a high accuracy. Furthermore, “Label” represents the information on the behavior being filmed in the video and shows that each behavior can be expressed using numbers (Table 4).

**Table 4:** Definitions of the keypoints, used for pose extraction, and behavior class number.

	Keypoint		Behavior class	
	0	Nose	0	Eating
	1, 2	Eye	1	Taking medicines
	3, 4	Ear	2	Drinking
	5, 6	Shoulder	3	Using a gas stove
	7, 8	Elbow	4	Cleaning the room
	9, 10	Wrist	5	Cleaning the furniture
	11, 12	Hip	6	Hanging laundry
	13, 14	Knee	7	Using a remote
	15, 16	Ankle	8	Reading
			9	Using a phone
			10	Smoking
			11	Talking
			12	Lying

### Algorithm for judging the behavior of older adults

Posec3d is an algorithm for action classification during action recognition using skeleton coordinates; that is, it is an algorithm that classifies the entire image into a single class. However, it is difficult to use in its original form, because older adults’ daily behaviors are too complex to be detected (i.e., the detection of what behavior is performed at a specific time on the basis of daily routine). Therefore, 90 frames were defined as the time to act, and the problem was mitigated by repeating Posec3d after every five frames. Using this Posec3d algorithm, action detection was performed every five frames to detect the actions of the older adults, and then the actions and respective corresponding time were coincidently recorded in the database. According to the set number of frames per second, the video was divided into image data. For example, based on 25 fps, five images correspond to 0.25 s, and 250 images corresponded to 10 s. Therefore, time information was derived, which allowed us to record the respective start times and end times of the older adult’s activities, configure a database, and derive information on what activities the older adult did at what time through the database. Using this process, the repeated daily behaviors of the older adults could be identified and implemented as the baseline data for ADLs through the stored database.

### Statistical analysis and experiment

**Figure 5:**
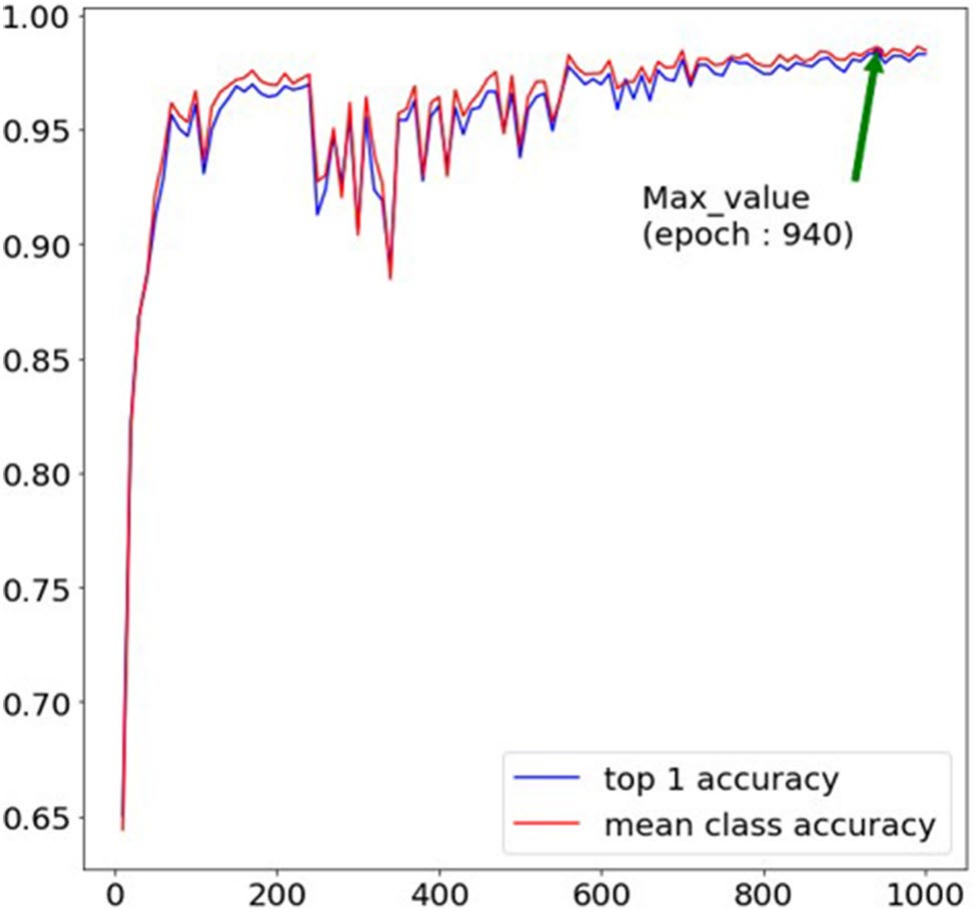
Result of training Posec3d. Based on the highest accuracy, the model with highest epoch (940) was selected as the final model.

To evaluate the accuracy of the machine learning and deep learning models, we utilized performance indicators such as accuracy, precision, recall, and F1-score. Accuracy is the ratio of the number of true positive and true negative to the total number of predictions. Precision means the ratio of true positives to true positives and false positives. Recall is calculated as the ratio of true positives to true positives and false negatives. The F1-score is calculated as the harmonic mean of precision and recall. The data used in this study showed some mild imbalance, and thus, we aimed to achieve an F1-score of more than 95%. In this experiment, we employed the Ubuntu 18.04 LTS operating system for machine learning using two RTX3090. The total batch size was set to 64, the learning rate was set to 0.025–1000 epochs, and the stochastic gradient descent was used as the optimizer function. In this case, cross entropy was used as the loss function. The model calculation of such a learning process was verified using the test annotation every 10 epochs, and the final model yielded the highest accuracy of older adults’ target behaviors. Specifically, among the 100 validations out of 1000 epochs, the 940-epoch model was selected as the best performing model as depicted in Fig. 5.

### Research ethics

The experimental data for verification were approved by the institutional review board of Dongguk University (DUIRB-202106-15). All the methods were carried out in accordance with the relevant guidelines and regulations. Informed consent was obtained from all the subjects and/or legal guardians that their information/images will be included in an online open-access publication and paper. Additionally, informed consent was obtained from all the subjects for participation.

## Results

### Evaluation of the test dataset

**Figure 6:**
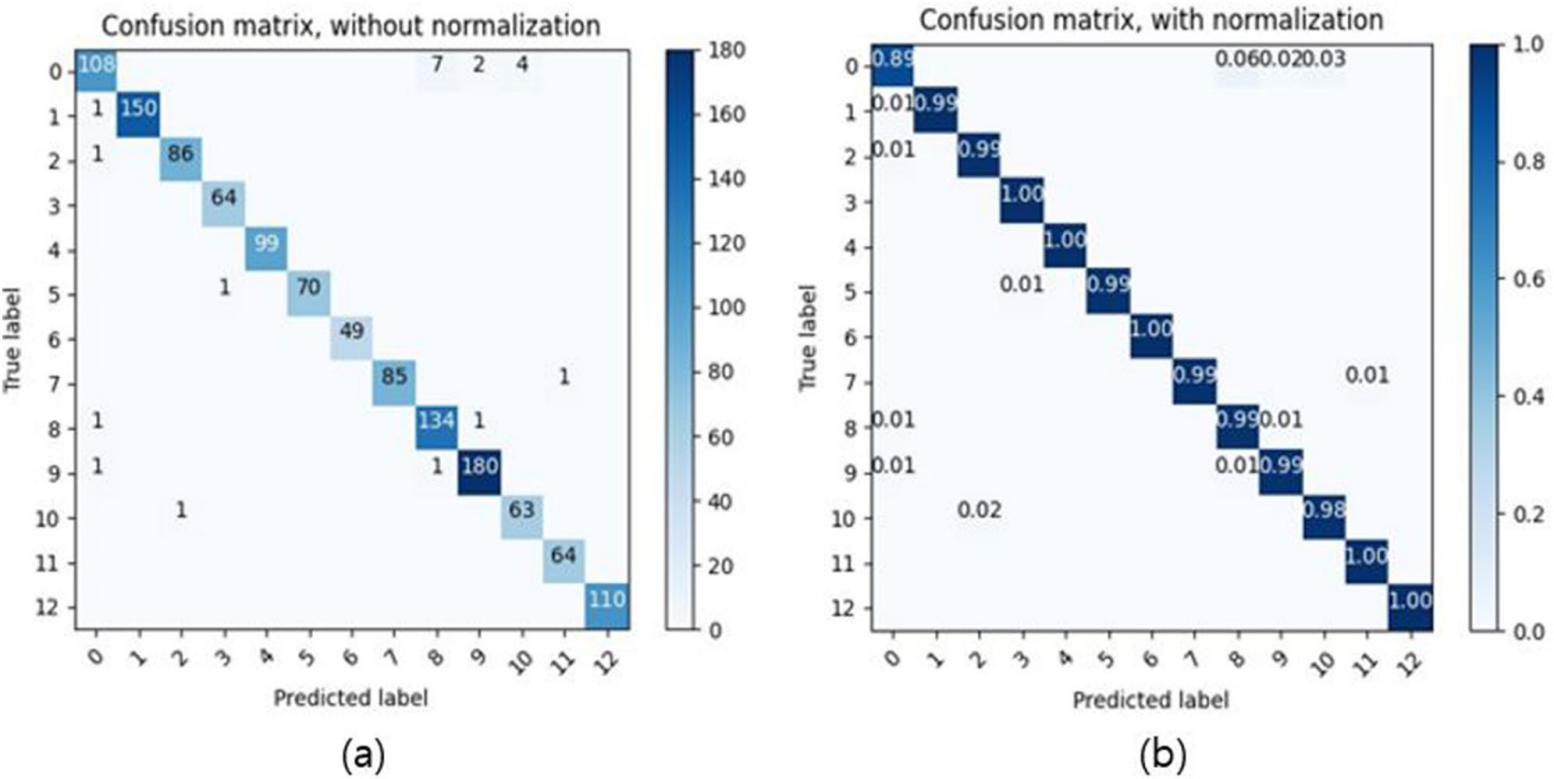
Confusion matrix of the results predicted by the behavior monitoring system. (**a**) Non-standard confusion matrix. (**b**) Standard confusion matrix according to the number of each class.

We obtained an excellent behavioral performance with 98% accuracy, 98% precision, 99% recall, and 98% F1-score, showing that all the indicators were accepted. In Fig. 6a, the actual label value and predicted label value are expressed using a confusion matrix. Since the number of labels for each class was not the same, as shown in Fig. 6b, we normalized the number of each class. The *x*- and *y*-axes in Fig. 6a,b represent the predicted and actual labels, respectively. Table 5 presents the performance indicators for each class.

**Table 5:** Performance indicators for each behavior class of the system.

Class	Action	Accuracy (%)	Precision (%)	Recall (%)	F1-score (%)
0	Eating	89	96	89	93
1	Taking medicines	99	100	99	100
2	Drinking	99	99	99	99
3	Using a gas stove	100	98	100	99
4	Cleaning the room	100	100	100	100
5	Cleaning the furniture	99	100	99	99
6	Hanging laundry	100	100	100	100
7	Using a remote	99	100	99	99
8	Reading	99	94	99	96
9	Using a phone	99	98	99	99
10	Smoking	98	94	98	96
11	Talking	100	98	100	99
12	Lying	100	100	100	100

The average F1-score was 98%, and the performance of 95% or more of the F1-score was established for a reliable behavior detection. However, as evident from Table 5, all the behaviors are not well detected. The precision of “Eating” is 96%, but recall is 89%, which is inferior to the performance of the other behavior classes. Figure 6 reveals that “Eating” is falsely detected the most as “Reading,” because the action of reaching for food and the action of grabbing a book show similar arm movements. Most of the behaviors are standing or sitting positions, but class 12 “lying” is a lying position, which is different from the other behaviors. As a result, “lying” detection is excellent with 100% accuracy, precision, and recall.

### Application of real data

**Figure 7:**
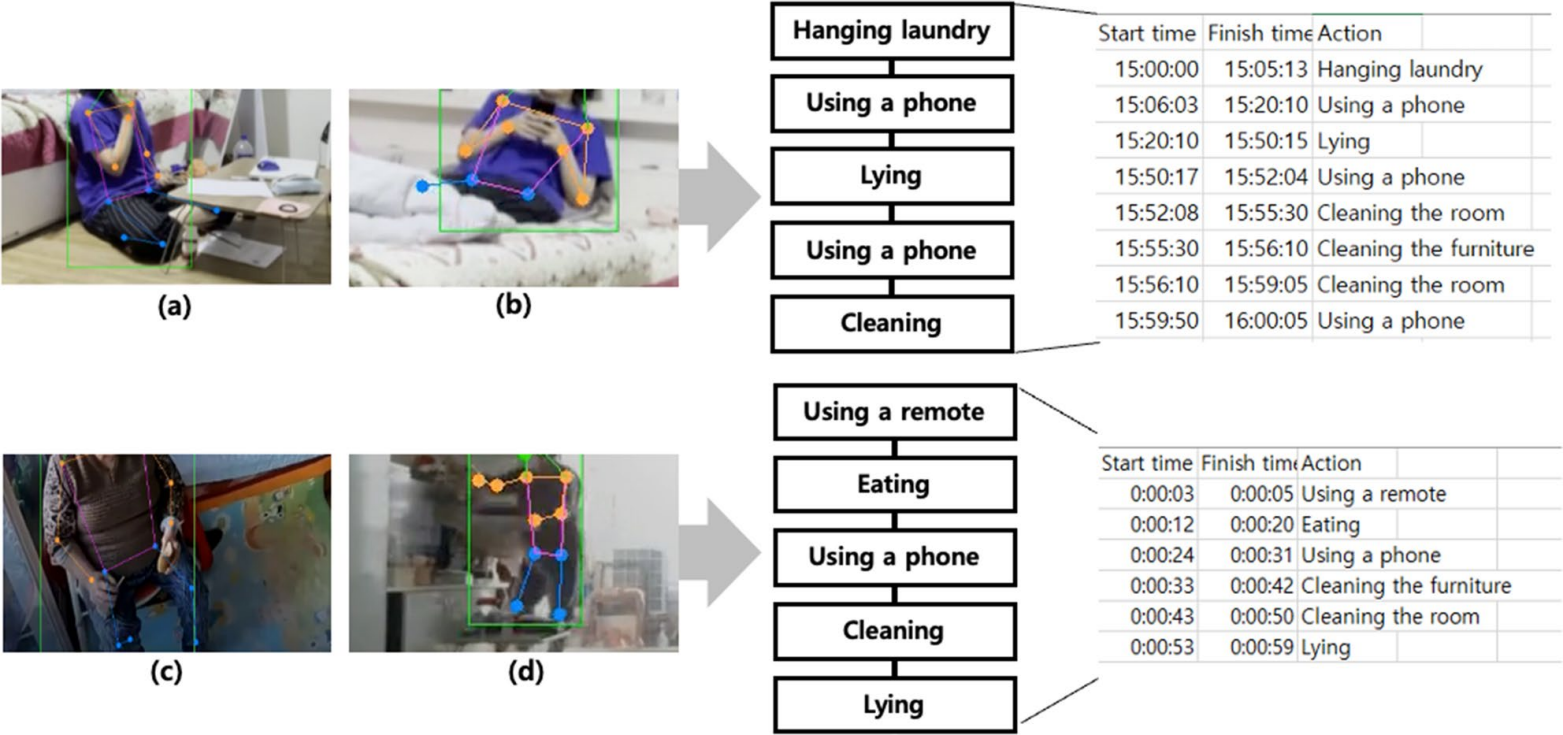
Application of the proposed method to monitor the behavior of an actual older adult. (**a,b**) Validation through collected data. (**c,d**) Validation using online news data.

The model was applied to actual videos to verify whether the actions shown in them were properly predicted or not. As shown in Fig. 7, the model was applied in older adults’ actual residential space to test and confirm that their actions were recorded in the database. In addition, it was applied to various existing videos to verify its workability in a real environment. The data shown in Fig. 7a,b were collected in a real environment, and permission was obtained from the person in the video to use the data. The data shown in Fig. 7c,d were published on YouTube^38^. The upper data shown in Fig. 7a,b could be transformed as a behavioral database, because the actual shooting time was 15:00. However, the data shown in Fig. 7c,d could not be transformed, because the actual time was unknown from the news. For this reason, the database was built to display changes in behavior in seconds by quickly replaying the human videos in the news. As shown in Fig. 7, when a video is given, the position of the person in the video and the skeleton coordinates of the human are detected. When the images shown in Fig. 7a,b were applied to the proposed system, some cases of false detection were obtained. For example, “Using a phone” was falsely detected as “Using a remote.” In this case, the database was designed in such a way that this behavior was identified as falsely detected as a different behavior for a short time, compared with the three detected behaviors before and after, and removed. Therefore, the behaviors could be organized into a database as shown on the right side of Fig. 7. These results confirm that various behaviors defined as intrinsic health risks were successfully detected and demonstrate the feasibility of long-term monitoring using the proposed system.

## Discussion

The focus of this study was to detect the various behaviors defined as chronic health risks in older adults with a high accuracy. Identifying such behaviors with a high accuracy will aid in detecting various daily life behaviors of an older adult in a dwelling, and long-term monitoring will become possible by storing the various behaviors in a database. Since older adults spend a lot of time at home, home is a valuable place for them and not just a place to live^46^. Therefore, we detected and identified their behaviors in a house.

Unlike the study of Yang and Hsu, who detected the rhythms of daily life in older adults, but not the exact actions/behaviors, our study objective was significantly different and comprehensive as it covered a wide range of intrinsic health behaviors of older adults using the proposed monitoring system^41^. Awais et al. established a monitoring system for ADL evaluation, but detected only four behaviors (sit, stand, walk, and lie down), reporting an F1-score of 87.2%, which is higher than that obtained in our case^42^. In contrast, Matsui et al., who detected only five behaviors, reported an F1-score of 40.7%, which is lower than that obtained in our study^43^. When a small number of behaviors are detected, building and utilizing a database through behavior detection of older adults are severely limited, because any unlearned behavior cannot be detected in this case. In this study, we overcame these limitations and performed detection for 13 different behaviors, achieving an average F1-score of 98%. Through proposed monitoring, it is possible to perform ADL evaluations, which can reduce the hassle of the observer. This study is a foundational research focused on the design and development of a long-term monitoring system for behavior identification and to realize risk aversion for older adults. After establishing a database following long-term monitoring using the proposed system, it is possible to derive the main behavioral patterns of the older adults in a house. Further, it is possible detect abnormal behaviors through the main behavioral patterns, and detect diseases such as dementia^47^. These systems are telemedicine systems that can manage the health of older adults and improve their quality of life in a non-invasive way.

## Conclusions

We used a Posec3d-based algorithm that performs deep-learning-based action recognition to develop an older adult monitoring system, which can monitor older adults living alone in a residential setting. Such a monitoring system organized and stored actions in a database depending on the start time and end time of certain actions. In particular, older adults’ behaviors that can cause social isolation, self-neglect, and health deterioration were defined as intrinsic risks in this study. Accordingly, three behavior categories were defined: (1) behaviors that can assess the ability to perform ADL in daily life, (2) behavior that can be unhealthy in the long run, and (3) behaviors that indicate the social relationship of older adults. The ETRI-Activity3D database, which includes videos of older adults, was used. Compared with other behavioral data, this dataset shows distinctive physical features of older adults, such as a curved spine different from that in young adults. In this way, we could perform specific monitoring customized for older adults.

Various older adult data could not be used because of storage limitations. To overcome the lack of diversity in the study data, we will apply this study technology in real settings with older adults, including personal houses and institutional settings like nursing homes, in the next stage of this research. In this study, the human skeleton data, which contain only motion information, were used, and thus, similar actions could not be properly detected. Therefore, in future studies, action recognition should be performed using the RGB image data and skeleton data in multimodal forms. Moreover, more accurate and comprehensive action recognition studies need to be conducted to further solidify the health management of older adults living alone.

## Data Availability

RGB dataset for recognizing the daily behavior of older adults in a robotic environment provided by ETRI was used. The data provided by ETRI were obtained from 100 persons (50 older adults and 50 general adults), and there were a total of 55 daily behavior classes. When observing the behavior of older adults, 55 types of behavior classes were constructed based on frequent activities, and in this study, 16 classes taken by 10 males and 10 females were used and reconstructed into 13 classes. Anyone can download the ETRI-Activity 3D data after applying for data on the ETRI Nanum website (https://nanum.etri.re.kr/share/dhkim008/robot_environment2?lang=en_KR). We do not have the permission to share these data.
